# Human Action Recognition and Note Recognition: A Deep Learning Approach Using STA-GCN

**DOI:** 10.3390/s24082519

**Published:** 2024-04-14

**Authors:** Avirmed Enkhbat, Timothy K. Shih, Pimpa Cheewaprakobkit

**Affiliations:** 1 Department of Computer Science and Information Engineering, National Central University, Taoyuan City 32001, Taiwan; avirmed2010@gmail.com; 2 Department of Information Technology, Asia-Pacific International University, Saraburi 18180, Thailand

**Keywords:** action recognition, recognize musical notes, spatial temporal attention graph convolutional network (STA-GCN), morin khuur, deep learning

## Abstract

Human action recognition (HAR) is growing in machine learning with a wide range of applications. One challenging aspect of HAR is recognizing human actions while playing music, further complicated by the need to recognize the musical notes being played. This paper proposes a deep learning-based method for simultaneous HAR and musical note recognition in music performances. We conducted experiments on Morin khuur performances, a traditional Mongolian instrument. The proposed method consists of two stages. First, we created a new dataset of Morin khuur performances. We used motion capture systems and depth sensors to collect data that includes hand keypoints, instrument segmentation information, and detailed movement information. We then analyzed RGB images, depth images, and motion data to determine which type of data provides the most valuable features for recognizing actions and notes in music performances. The second stage utilizes a Spatial Temporal Attention Graph Convolutional Network (STA-GCN) to recognize musical notes as continuous gestures. The STA-GCN model is designed to learn the relationships between hand keypoints and instrument segmentation information, which are crucial for accurate recognition. Evaluation on our dataset demonstrates that our model outperforms the traditional ST-GCN model, achieving an accuracy of 81.4%.

## 1. Introduction

Recently, human action recognition has garnered substantial interest in machine learning tasks. The capability to identify actions from a sequence of frames in the video has numerous applications, including the interaction between humans and computers, intelligent monitoring through video, robot vision, multimedia, and hand gesture recognition by using hand keypoint and segmentation [1], recognition music sounds and generate musical compositions [2].

The recognition of hand gesture techniques can be broadly classified into two types: image-based and skeleton-based. Image-based methods rely on sequences of RGB or RGB-D images as their input, while skeleton-based methods use 2D or 3D hand joint sequences. The application of deep learning techniques, including Long Short-Term Memory (LSTMs) and Convolutional neural network (CNNs) have been implemented for recognizing hand gestures by using hand skeleton sequences as input. However, the aforementioned methods fall short of maximizing the utilization of the spatial-temporal connections between joints.

Numerous techniques have been suggested to perform action recognition, such as skeleton-based recognition that utilizes the human body joint trajectories [3]. Skeleton data consists of the movements of human body joints and is a compact and effective means of recognizing actions [4]. It is also resilient to changes in the background, Graph Convolutional Networks (GCN) [5] to model the human body skeletons, MMPose for real-time skeleton detection and tracking [6], The American Sign Language (ASL) hand gesture recognition [7], which consists of two parallel convolutional neural networks, one processing spatial features and the other processing motion features. The spatial CNN takes a single image of a hand gesture as input and extracts spatial information, while the motion CNN takes a sequence of such images and extracts motion information. Both CNNs consist of several convolutional and pooling layers, followed by one or more fully connected layers. The outputs of the two CNNs are then combined and passed through a softmax layer to obtain the final gesture classification result. Sanchez-Caballero et al. [8] implemented a real-time method for recognizing human actions from raw depth video sequences using Recurrent Neural Networks (RNNs).

The recognition of human actions in computer vision is a challenging task due to the time dimension and the complexities of action speed across frames, human pose, and distractions in video sequences. This is particularly challenging when it comes to recognizing the beat and notes of music in videos. 

To address this challenge, we introduce a human-computer interaction system that can recognizing musical notes during musical performance. This system can recognize dynamic finger movements, the interaction between the musician and the instrument, as well as the distinct sound of the instrument. In our methodology, we employ data pre-processing, involving the MMPose method for pose estimation and the YOLOACT segmentation method to identify different parts of musical instruments. For action recognition, we leverage a Graph Convolutional Network with spatial-temporal attention (STA-GCN), which integrates both spatial and temporal attention mechanisms. The model employs a graph convolutional network to learn pose or spatial features from skeletal hand joints and a temporal convolutional network to capture motion dynamics in the hand movement sequence. The attention mechanisms fuse spatial and temporal features to enhance recognition accuracy, with the pose and motion streams sharing the same network architecture but processing different input data. To extract features, STA-GCN first initializes the skeleton graph and then applies spatial-temporal graph convolution, along with spatial and temporal graph attention. These features are then passed through a temporal pyramid pooling layer (TPP) to obtain multiple scale temporal features. Finally, a fully connected layer and Softmax function are utilized for hand gesture recognition. The recognition outcome is acquired by concatenating the extracted features of both the pose and motion streams. The main contributions are summarized as follows:

- We propose an architectural framework for simultaneous action and musical note recognition in music performances. This framework leverages a multimodal approach, combining visual (RGB and depth) data with motion capture data.
- The proposed method utilizes MMPose and YOLOACT for data pre-processing, and a Spatial Temporal Attention Graph Convolutional Network (STA-GCN) for recognizing notes as continuous gestures.
- We introduce a new dataset specifically designed for Morin khuur performances, enriching the existing resources for musical instrument recognition tasks.

The structure of the remaining manuscript is outlined as follows: Section 2 introduces the related work, Section 3 presents the system architecture of the proposed method, Section 4 demonstrates the experimental results; and Section 5 concludes our work.

## 2. Related Work

Advancements in Human Action Recognition (HAR) have been driven by the emergence of deep learning architectures. These architectures surpass traditional methods that rely on hand-crafted features, which ignore the semantic connections between human joints [9]. Deep learning emphasizes the significance of understanding the semantic human skeleton for accurate action prediction. Recurrent Neural Networks (RNNs) [10] offer a direct method to represent skeleton data as a sequence of coordinate vectors, with each vector denoting a specific human body joint. RNNs sequentially encode temporal information, capturing the dynamics of body movements over time. Convolutional Neural Networks (CNNs) [11] dramatically improve skeleton-based action recognition by analyzing both body joint positions (spatial) and their movement over time (temporal) together. This eliminates complex pre-processing and lets CNNs automatically learn action details, making them powerful tools for understanding human movement from skeletal data. For instance, DD-Net [12] utilized 2D human skeleton data within a lightweight CNN architecture to encode body joint movements and improve action recognition. Liu et al. [13] introduced ConvNeXts as a challenge to the dominance of Vision Transformers (ViTs) in image classification, achieving competitive accuracy and scalability by leveraging grouped convolutions and efficient channel interactions. However, ConvNeXts involve a high number of parameters and require substantial labeled data for optimal performance. CondenseNet [14], inspired by DenseNet, utilizes learned group convolutions to reduce computations, resulting in smaller models and faster processing, but this comes at the cost of additional complexity. Similarly, Yulin et al. [15] introduced dynamic transformers for efficient image recognition, addressing the limitations of fixed-size image embeddings by dynamically adapting the number of tokens based on image complexity. On the other hand, this approach may increase computational overhead during inference due to dynamic grid resizing.

In parallel, pose estimation techniques have also witnessed significant advancements, contributing to the improvement of HAR systems. Pose estimation plays a crucial role in extracting skeletal information from human movements, providing valuable cues for action recognition algorithms. Recent works such as PoseFormerV2 by Zhao et al. [16] This model utilizes the frequency domain to represent human skeleton sequences, boosting the accuracy and stability of 3D human pose estimation. Despite its effectiveness in discerning intricate spatial and temporal features, the model may face computational complexity challenges. Additionally, while the paper focuses solely on skeletal data, integrating RGB or depth information with PoseFormerV2 could enhance pose estimation robustness in future research. Similarly, to TokenPose, introduced by Li et al. [17], the model encodes each keypoint (body joint) as a token. This allows the model to simultaneously learn the visual cues and the relationships between different body parts. Despite these advancements, pose estimation techniques may still face challenges in accurately capturing complex human movements, especially in scenarios with occlusions or limited visibility.

Furthermore, advancements in object detection and instance segmentation have facilitated the localization and tracking of human actions in videos. Techniques like BoxInst [18] achieve this by leveraging only bounding box annotations during training. Unlike traditional methods that require both bounding boxes and masks, BoxInst demonstrates impressive accuracy and efficiency instance segmentation tasks. However, a limitation of this method is its reliance on box annotations, which may not always provide sufficient information for accurate segmentation, especially in complex scenes with overlapping objects or fine-grained details. Additionally, the performance of BoxInst could be impacted by the quality and consistency of the box annotations. RefineMask proposed by Zhang et al. [19] refines instance segmentation by iteratively improving mask predictions with fine-grained features. This achieves high-quality segmentation but may suffer from high computational complexity, especially during the refinement stage, requiring significant resources and time. Lee et al. [20] introduced a CenterMask method for real-time instance segmentation that eliminates the requirement for anchor boxes, thus bypassing pre-defined shapes for object detection. The approach, CenterMask, prioritizes predicting object centers and subsequently refining those predictions with segmentation masks. Achieving notable speed and accuracy, CenterMask simultaneously predicts object centers and segmentation masks. However, a constraint of CenterMask lies in its dependence on anchor-free techniques, potentially leading to diminished performance in scenarios involving highly overlapping or irregularly shaped objects.

Recent works, such as AdaDet proposed by Yang et al. [21], have introduced adaptive object detection systems that leverage early-exit neural networks. The model dynamically adjusts its inference process based on input complexity, allowing for faster predictions with minimal sacrifice in accuracy. However, a limitation of AdaDet lies in its reliance on early-exit neural networks, which may require additional computational resources for training and inference compared to traditional object detection systems.

While existing methods might neglect the inherent connections between joints (skeleton topology) or suffer from complex design processes, graph-based methods have emerged as strong contenders in achieving high accuracy on popular benchmarks. Inspired by the natural structure of the human body, graph-based approaches have recently shown impressive results. Notably, ST-GCN [22] pioneered the use of graph convolution operations alongside temporal convolutions to simultaneously model both spatial and temporal information within the skeleton data. To further enhance the flexibility of the graph topology itself. For instance, Wang et al. [17] utilized a graph to represent human joints and applied the Spatial Temporal Graph Convolutional Network (ST-GCN) for feature extraction. The ST-GCN consists of multiple Graph Convolutional Network (GCN) blocks, which effectively grasp the structural details of the human body, as bone structure information is naturally organized as a graph connecting major points in the human body. However, GCN approaches can have heavy computational overhead. This can result in slower inference times and increased resource requirements, limiting their practicality in real-time applications or resource-constrained environments. 

The MMPose model [6] is introduced to enhance the efficiency of ST-GCNs for real-time human skeletal posture estimation. In MMPose, Graph Convolutional Transformers (GCTs) are implemented as a replacement for the traditional graph convolutions used in ST-GCNs. By leveraging the self-attention mechanism of transformers, GCTs effectively capture long-range dependencies. This enables MMPose to capture global context and refine the spatial-temporal representations of poses [17]. Additionally, MMPose incorporates a hierarchical graph structure that captures multi-scale dependencies. This approach involves incorporating multiple graph levels with varying resolutions, allowing the model to effectively capture both local and global dependencies. Consequently, the representation and understanding of human poses are improved. One drawback of the MMPose model is its higher complexity compared to traditional pose estimation models. This increased complexity can lead to longer training times. Furthermore, the added complexity of MMPose raises the risk of overfitting, where the model becomes too specialized to the training data and may not generalize well to unseen data. 

On the other hand, in the realm of action recognition, the demand for incorporating spatial information alongside temporal patterns is growing. Algorithms like YOLACT [23] offer a solution by providing precise object segmentation masks, enabling the extraction of spatial information crucial for a localized understanding of actions. Integrating YOLACT can significantly improve the precision of action recognition tasks and enhance scene analysis. Lin et al. [24] proposed a system that leverages YOLACT++ for precise human body part segmentation and identification. This information is then combined with the feature extraction capabilities of ResNet18, allowing the system to learn distinctive features and achieve accurate posture classification. However, the system’s performance is highly dependent on the quality, size, and diversity of the training data. Limited, biased, or insufficient training data can lead to decreased recognition accuracy or hinder the system’s ability to generalize well to different people or pose variations.

Recent trends in HAR research emphasize the importance of multi-task learning and cross-modal fusion techniques. Integrating information from multiple modalities such as RGB images, depth maps, and motion data allows for a more comprehensive understanding of human actions and gestures. For instance, the work by Blanco et al. [25] proposed a method for violin performance analysis that integrates motion capture data and audio signals, enhancing the robustness and accuracy of action recognition in musical contexts. However, the study’s focus on a randomized trial design may limit the generalizability of the findings, as individual learning styles and preferences could influence the effectiveness of the feedback mechanism.

Our proposed approach aims to bridge this gap by leveraging deep learning techniques to simultaneously recognize actions and musical notes in Morin khuur performances. This approach has the potential to make significant contributions to both music technology and human-computer interaction research. 

## 3. Proposed Architecture

**Figure 1:**
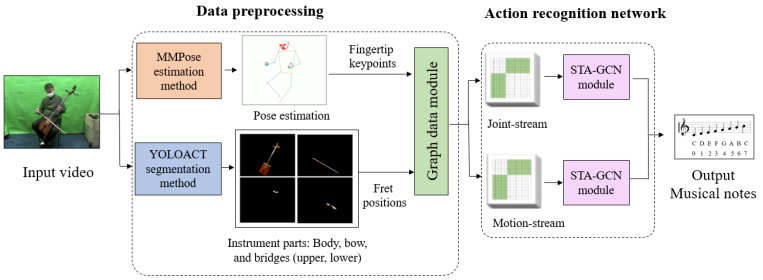
Our proposed architecture.

In our proposed architecture, we feed video as input. The data preprocessing stage involves utilizing the MMPose method for pose estimation and the YOLOACT segmentation method for identifying the instances of various instrument parts, such as the body, bow, upper bridge, and lower bridge. The action recognition process involves the use of STA-GCN for detecting and classifying actions from skeletal data. This action recognition process employs a two-stream architecture for recognizing hand gestures from skeletal data, with the joint stream and motion stream being generated from hand keypoints and frets location on the fingerboard, as depicted in Figure 1. The model will ultimately output the prediction of musical notes.

### 3.1. MMPose Method

MMPose [6] is a real-time human skeleton detection and tracking method for multi-person pose estimation that supports a wide range of features such as hand, whole-body, pose, 2D keypoints, 3D mesh reconstruction, and 3D surface. It is a state-of-the-art method that achieves high accuracy and efficiency by leveraging multi-scale feature learning, soft-argmax-based keypoint aggregation, and a fully-convolutional network architecture. Additionally, MMPose incorporates data augmentation techniques and is trained using a combination of supervised and unsupervised learning methods. Overall, MMPose is a powerful tool for accurately and efficiently estimating poses across multiple individuals with diverse features. The model utilizes the High-Resolution Representation Network (HRNet) [23] as a backbone, which is capable of preserving high-resolution representations throughout the entire process.

**Figure 2:**
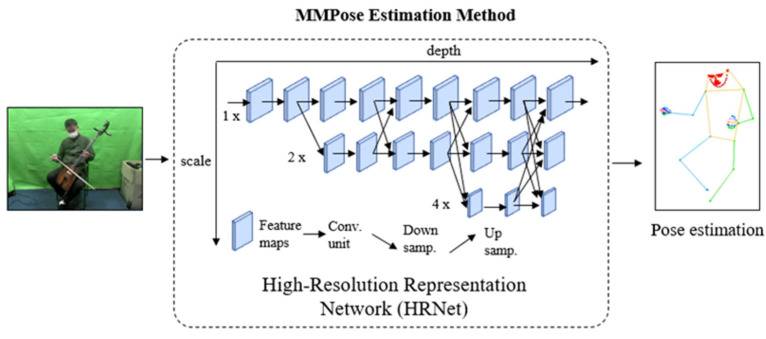
The HRNet architecture in the MMPose estimation method.

The HRNet utilizes the concept of multi-resolution representations, where high-resolution representations are extracted and fused to generate more robust features. HRNet has a unique architecture that enables it to process high-resolution images efficiently, by avoiding down-sampling or up-sampling operations that may cause information loss or computation overhead. It has multiple stages that gradually refines features, allowing it to learn increasingly complex representations. The HRNet architecture starts from high-resolution sub-network, followed by the addition of low- resolution sub-network as the network goes deeper to create multi- resolution. The multi- resolution information is fused by exchanging information repeatedly across parallel multi-resolution sub-networks. Finally, the HRNet estimates keypoints based on the high-resolution representations that are outputted. Figure 2 displays the HRNet network in the MMPose estimation method, where the x-axis indicates the network depth and the y-axis represents the feature map scale.

**Figure 3:**
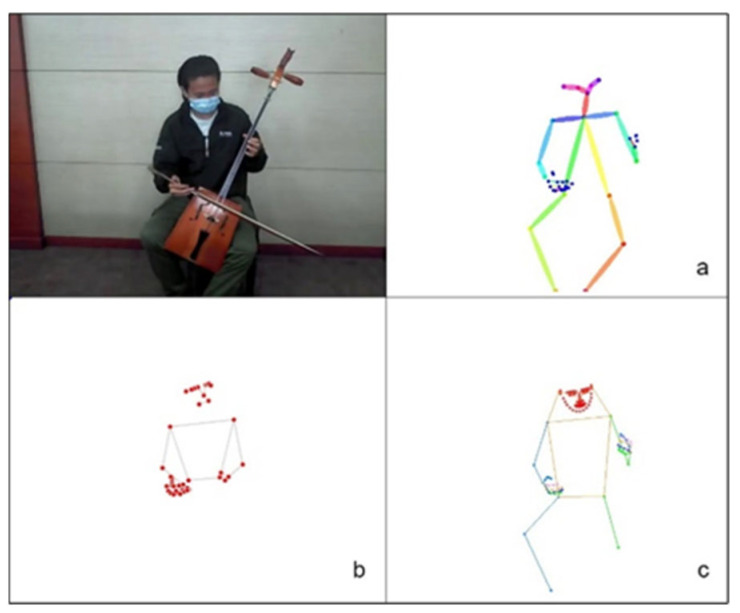
The whole-body keypoints estimation results of MediaPipe (**a**), OpenPose (**b**), MMPose (**c**).

We utilize the whole-body top-down pose estimator provided by MMPose as a pretrained model to estimate the 133-point keypoints of the entire body from RGB videos and create a graph of finger keypoints. The whole-body human pose estimation method has been outperformed in terms of both robustness and efficiency. We compared the whole-body keypoints estimation from human pose with MediaPipe, OpenPose, and MMPose. The results shown that MMPose method is outperform other methods as shown in Figure 3.

We compared the whole-body keypoints estimation from human pose performance with MediaPipe, OpenPose, and MMPose in term of Frame per second (FPS), number of incorrect keypoints prediction (Incorrect), and number of missing keypoints (Missing) as shown in Table 1.

Our evaluation of 100 video frames revealed MMPose to be the superior choice. It achieved a processing speed of 4 FPS with the lowest number of both incorrect (0) and missing keypoints (0). This combination of speed and accuracy is essential for our task, as precise keypoint detection and real-time performance are critical for capturing the intricate hand movements and instrument interaction that characterize Morin khuur music performances.

In contrast, MediaPipe exhibited the fastest processing speed (12 FPS) with a significant number of incorrect keypoint predictions (16) and missing keypoints (43). OpenPose, while achieving a processing speed of 1 FPS, suffered from even more incorrect keypoint predictions (24) and missing keypoints (9).

### 3.2. YOLACT Segmentation Method

**Figure 4:**
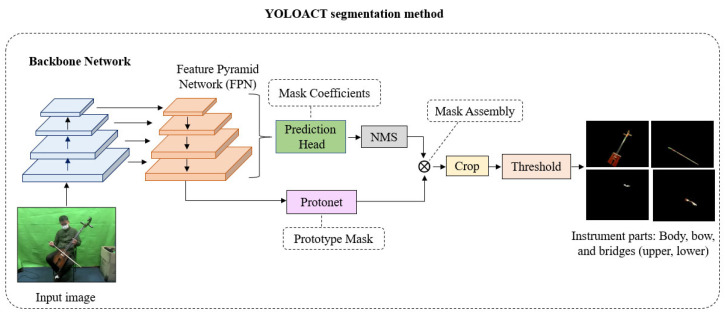
YOLACT segmentation method.

YOLACT is a real-time instance segmentation algorithm developed by Bolya et al. [26]. It is capable of detecting and segmenting objects in an image, where each object is labeled with a unique mask. YOLACT has implemented a prototype generation network that responsible for learning a set of object features used to create a set of prototype masks. These prototype masks are then used to compute a set of feature maps for performing instance segmentation. To improve the accuracy of instance segmentation, a fusion module is utilized. This module combines information from the prototype masks, feature maps, and class labels to produce a final set of instance masks. Additionally, the loss function is designed to promote the learning of precise object masks and effective object prototypes. It combines a semantic segmentation loss, a mask prediction loss, and a prototype similarity loss. YOLACT segmentation method is shown in Figure 4.

We employ the ResNet101-FPN network as the backbone architecture for feature extraction from an image, accomplished through a series of convolutional layers. These convolutional layers are responsible for acquiring diverse image features, encompassing elements like edges, textures, and shapes. We augment ResNet-50 with the FPN, as it has the capacity to enhance the model’s efficacy in object detection and segmentation tasks.

**Figure 5:**
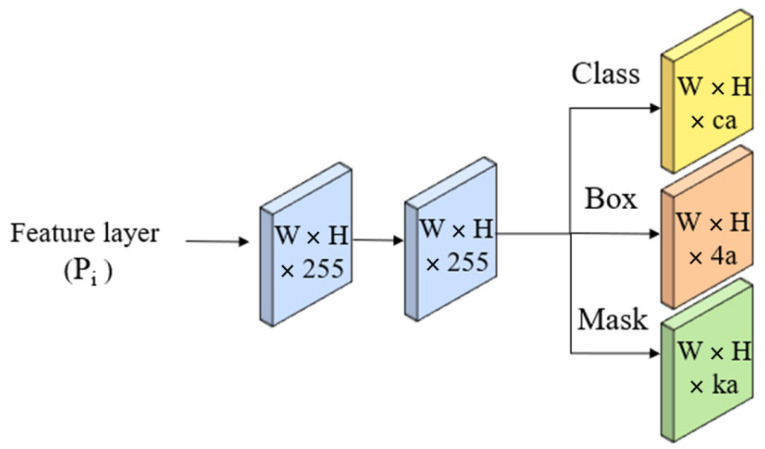
Prediction Head.

The prediction head is a fully convolutional network that takes the feature pyramid from the FPN as input and predicts the class probabilities, bounding boxes, and mask coefficients for each object as shown in Figure 5.

In this context, *c* stands for the number of classes, *a* denotes the anchors for feature layer *P_i_*, and *k* signifies the prototypes.

**Figure 6:**
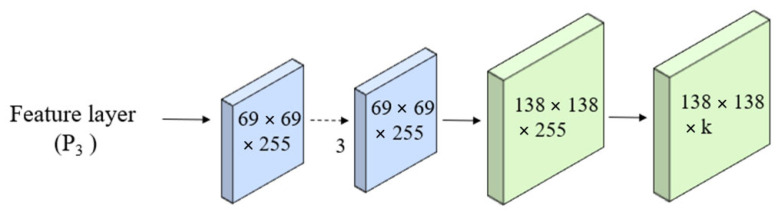
Protonet network.

Protonet is a network that predicts a set of prototype masks for the entire image. It learn during training to represent the different object categories that the model can detect and segment objects in real-time. We employ the Feature Pyramid Network (FPN) within the Protonet network. Importantly, the deeper backbone features produce better performance on smaller objects. In our model, the largest feature layers, denoted as *P*_3_, are the deepest. The increase in size is accomplished through an upsampling operation, followed by a convolutional layer, and further enhanced by the ReLU activation function. The architecture of the Protonet network is depicted in Figure 6. The labels provide information about feature dimensions and channels corresponding to the image size. The arrows represent 3 × 3 convolutional layers, with the exception of the final convolutional layer, which is 1 × 1 in size and is denoted as *k* to represent prototypes.

Mask coefficients are learned during the training process and are used to weight and combine the prototype masks to generate instance-specific masks for objects detected in the image. Essentially, they help determine the shape and appearance of the mask for each individual object, allowing for accurate instance segmentation. After the prediction head has computed mask coefficients for each object, Non-Maximum Suppression (NMS) is employed to eliminate redundant bounding boxes among the predicted ones, ensuring each object is detected only once. Subsequently, instance masks are generated through Mask Assembly. These masks are then extracted for each object using the crop operation, and finally, the threshold operation is applied to binarize the instance masks for each object. Then generate segmentation masks for the instrument parts, such as the body, bow, lower bridge, and upper bridge.

Mask Assembly is used to generate instance masks. We combine the outputs of the prototype branch and the mask coefficient branch by using a linear combination of the former with the latter as coefficients. Subsequently, we apply a sigmoid nonlinearity to obtain the final masks. These procedures can be implemented through a single matrix multiplication followed by a sigmoid function, as shown in Equation (1) [26].

(1) $$M\;=\sigma \;(PC^{T})$$

In this context, *M* is the predicted mask for the object, *σ* is the sigmoid function, *P* represents a matrix of prototype masks with dimensions *h × w × k*, with their corresponding coefficients *C*, and *T* is the matrix transpose.

### 3.3. Graph Data Module

**Figure 7:**
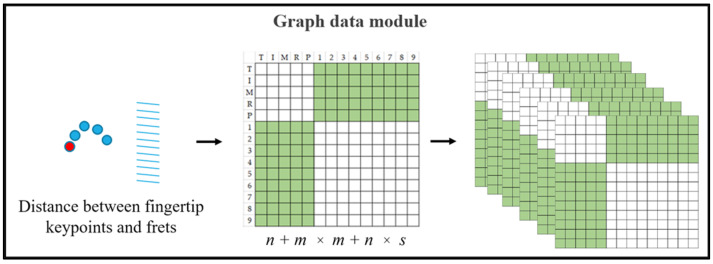
Graph data module.

The MMPose estimation method generates pose estimation data, from which we extract fingertip keypoints. These keypoints, along with fret positions obtained from instrument parts using the YOLOACT segmentation method, are then used as inputs for the Graph Data module. This module calculates the distances between fingertip keypoints and fret positions, as illustrated in Figure 7. The resulting joint-stream and motion-stream graph data is subsequently fed into the STA-GCN module for further processing.

Each green element in Figure 7 represents the distance between a fingertip keypoint and a fret position across a video sequence. White elements represent empty values. Fingertip keypoints, denoted by *n*, correspond to the total number detected by the MMPose estimation method. Similarly, fret positions, denoted by *m*, represent the total number of frets on the Morin khuur instrument. Additionally, the sequence length, indexed by *s*, has a value of 30 in this study.

The distance computation involves considering all possible combinations of fingertip keypoints and fret positions throughout the entire sequence. This calculation results in *n* + *m* × *m* + *n* × *s* calculations, effectively capturing the relationships between every fingertip and every fret position across each frame in the sequence.

#### Fret Positioning Calculation

**Figure 8:**
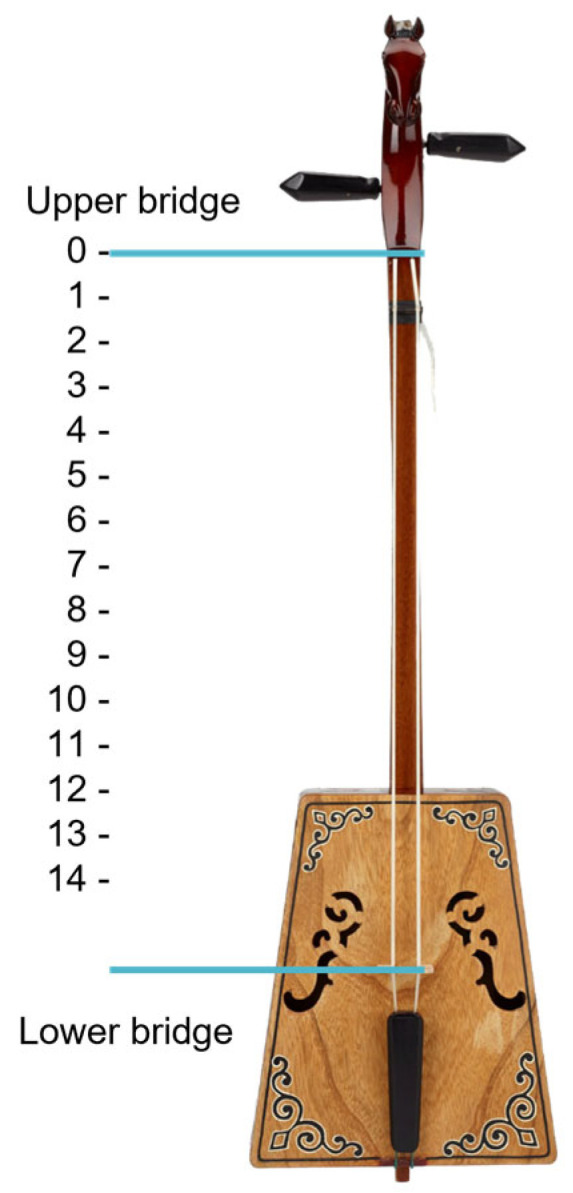
The scale length of the instrument.

The morin khuur, a traditional Mongolian bowed string instrument, does not have frets on its bridge. Unlike many other string instruments, such as guitars and lutes, the Morin khuur has a smooth, unfretted neck. To determine fret positions, the space between frets decline in a consistent ratio, as depicted in Figure 8. Table 2 illustrates the standard scale length for each fret based on the size of the instrument, which includes small, medium, and large sizes [27].

In a musical performance video, defining the notes presents a challenge due to variations in instrument size, which depend on both the camera’s recording distance and the inherent size of the instrument. Therefore, it becomes essential to establish the scale length of each fret, representing the distance between the nut and the bridge when the string is pressed down. The formula used for calculating the scale length of frets on stringed instruments is given in Equation (2).

(2) $${\mathrm{SL}}_{i}=\frac{\mathrm{SSL}}{\mathrm{SI}}$$

In this context, *SL_i_* denotes the scale length of fret *i*, where *SSL* stands for the standard scale length, which is determined from a table based on the instrument size and fret index. *SI* represents the size of the instrument, with *i* denoting the fret index ranging from 1 to 14. Additionally, *L* represents the length of the fretboard for each instrument size: 56.6 cm for large size, 54 cm for medium size, and 5.4 cm for small size. 

As an example, let’s calculate the scale length of fret 1 for a large-sized instrument (56.6 cm). The standard scale length value in the Table 2 for fret index 1 of a large-sized instrument is 6. Applying the formula, the result is:

$${\mathrm{SL}}_{1}=\frac{6}{56.6\;}\;\approx \;0.106$$

### 3.4. Action Recognition Network

We have specifically designed the Action Recognition Network to recognize the relationship between Morin khuur instrument positions, finger positions, and to link them to actual music notes. This network leverages a combination of Spatial-Temporal Attention and Graph Convolutional Networks (STA-GCN).

**Figure 9:**
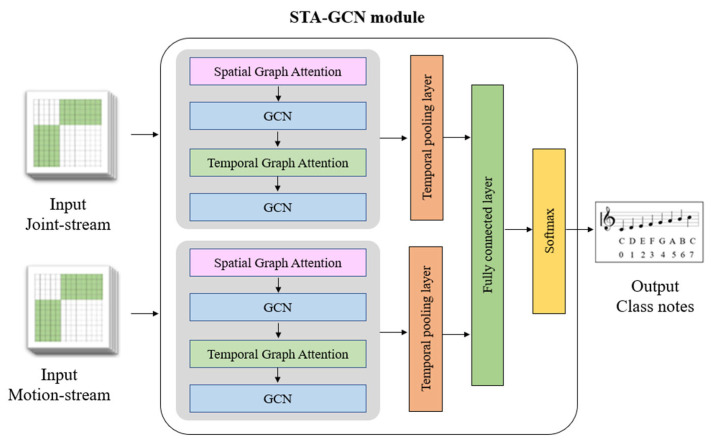
The Spatial-Temporal Attention and Graph Convolutional Network (STA-GCN) module.

Initially, the joint-stream and motion-stream graph data are input into the Spatial-Temporal Attention and Graph Convolutional Network (STA-GCN) module, as illustrated in Figure 9. Within this module, the spatial graph attention mechanism captures the spatial dependencies among the key points in the joint-stream and motion-stream data, allowing the model to learn how the relative positions of hand joints and the locations of instrument sections influence each other. Subsequently, the Graph Convolutional Network (GCN) refines the learned features for each data stream through graph-based convolutions. Imagine the hand and instrument segmentation data as graphs, with keypoints/instrument sections as nodes and connections between them as edges. The GCN leverages these connections to further refine the understanding of how these elements relate to each other within each stream.

Next, the module integrates temporal graph attention to capture temporal relationships between consecutive frames in the input data, thereby enhancing the understanding of the dynamic evolution of the pose and motion information over time during a performance. The features extracted through spatial and temporal attention mechanisms are then passed through a temporal pooling layer, which aggregates information across frames, capturing the overall movement patterns. The extracted features are subsequently processed by a fully connected layer to capture high-level representations, followed by a Softmax layer for predicting musical notes based on the learned features.

By employing this two-stream architecture with separate attention mechanisms for both spatial and temporal information, the STA-GCN model effectively learns the intricate relationships between hand keypoints and instrument segmentation data, ultimately enabling it to recognize the connections between Morin khuur instrument positions, finger positions, and the corresponding musical notes being played.

### 3.5. Dataset

We collected data from both professional and novice musicians’ performances using two cameras: a Logitech C200 webcam (Logitech, Lausanne, Switzerland) for recording RGB footage (color video) and an Intel^®^ RealSense™ camera SR300 (Intel Corporation, Santa Clara, CA, United States) for capturing RGB-depth data, providing combined color and depth information. This resulted in a dataset containing RGB front-view and side-view videos of the performances, along with corresponding MIDI files (musical note information) and depth sequence data. To extract meaningful information from the videos, we applied preprocessing techniques using a segmentation module. This involved identifying key points on the musician’s body (landmarks) and segmenting the four individual parts of the Morin khuur (body, bow, upper bridge, and lower bridge) for further analysis.

**Figure 10:**
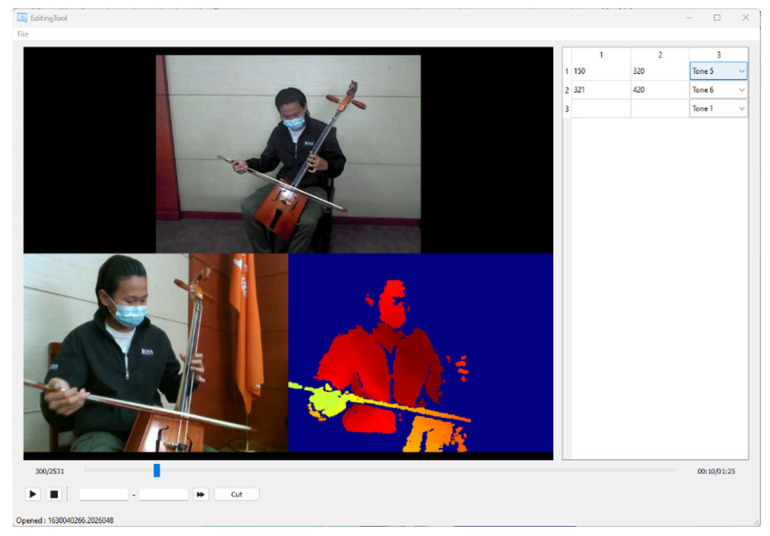
Data collection and editing tool.

**Figure 11:**
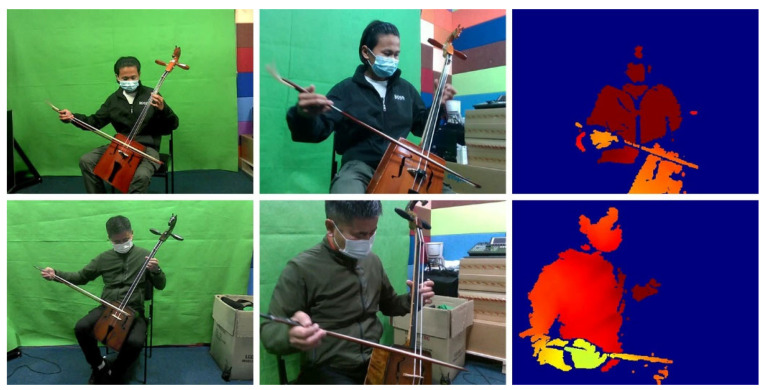
Data examples include RGB images captured from front and side views, along with depth images.

Due to the unique nature of Morin khuur gestures, we developed a custom data collection and editing tool (as shown in Figure 10). This tool streamlines data gathering by allowing for simultaneous recording of video and audio, editing and trimming of captured data across different types (RGB front-view, side-view, depth video, depth raw, and audio), generating an annotation file, and saving data in suitable formats for further processing, as illustrated in Figure 11. Examples of data include RGB images captured from front and side views, along with depth images.

The final dataset consists of 600 videos divided for training, validation, and testing purposes. Seventy percent of the videos are used to train the machine learning model, 15% are used for validation during training to optimize the model’s performance, and the remaining 15% are used for testing the model’s accuracy on unseen data. The dataset has been released and is available on the website, accessible through the following link: “https://drive.google.com/drive/folders/1WLiPbjJ4Y0UM6S6KH-2hJ6fClNEUL3IE?usp=drive_link (accessed on 10 April 2024)”.

The STA-GCN model was trained using the Adam optimizer, chosen for its efficiency in handling sparse gradients and noisy objectives. Hyperparameter tuning involved adjusting the learning rate (ranging from 0.0001 to 0.001) and varying the number of hidden units in GCN layers (e.g., 16, 32, 64). This process allows evaluation of the model’s capacity to learn complex relationships. Evaluation of the STA-GCN model typically employs standard accuracy and loss metrics for action recognition tasks, indicating the overall percentage of correctly classified musical notes.

## 4. Experimental Results

**Figure 12:**
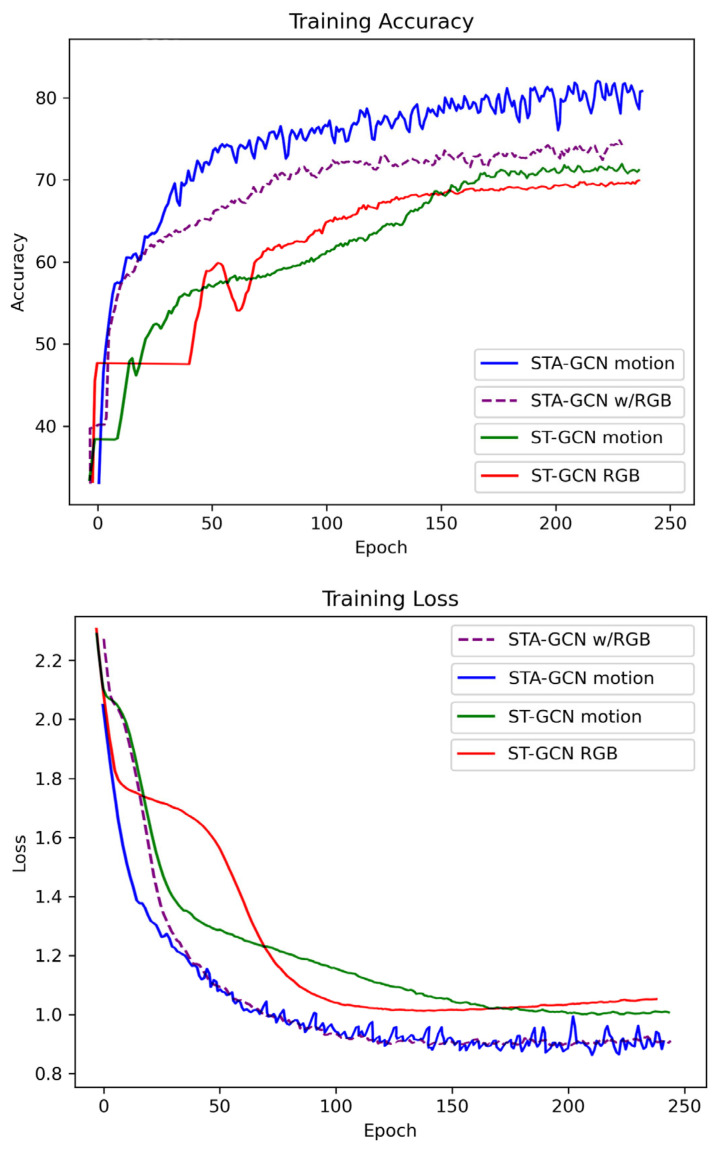
Illustrates the training accuracy and loss plot.

We evaluated the performance of our proposed method (STA-GCN) by comparing it with various data types, as illustrated in Table 3. These include two-camera data, data without side camera input, data without front camera input, motion data, motion data combined with RGB, and RGB data alone, as well as ST-GCN with motion data and ST-GCN with RGB as baseline models. The results indicate that our proposed method utilizing motion data achieved the highest accuracy of 0.81, with a loss value of 0.93. Conversely, the lowest accuracy of 0.43, with a loss of 2.76, was obtained when using two-camera data. The training accuracy and loss are illustrated in Figure 12. 

The reason behind this discrepancy is likely due to several factors. Motion data captures small details of musician movement, including finger motions and bow control, which are crucial for musical expression and note recognition. Camera data, especially in single-camera setups, might miss these intricacies due to occlusion or limited viewpoints. Additionally, a two-camera setup might not be optimal for capturing the most relevant information for note and action recognition. The camera angles might miss crucial hand and instrument interactions crucial for accurate recognition. RGB data alone might be insufficient for differentiating subtle movements related to specific notes, especially if lighting conditions or background variations are present. Finally, combining motion data with RGB data might not achieve the best results if the fusion is not effective. The specific way of combining these data streams could introduce noise or redundancy, negating the potential benefits of each type.

### Compare the Performance of the MASK-RCNN and YOLACT Methods in Segmenting Objects

**Figure 13:**
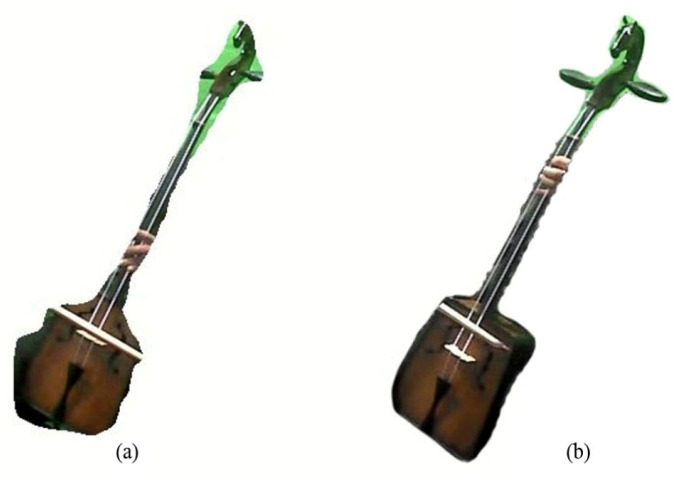
MASK-RCNN (**a**) and YOLACT (**b**) segmentation comparison.

Our evaluation of MASK-RCNN (a) and YOLACT (b) for instrument segmentation, illustrated in Figure 13 and Table 4, revealed YOLACT’s superior performance due to its refined backbone network, optimized anchor design, and swift mask re-scoring. This superior performance led us to adopt YOLACT for instrument segmentation in our paper.

Table 4 compares the segmentation performance of Mask R-CNN and YOLACT based on metrics including APmask and FPS. YOLACT achieves a higher APmask (32.6) and a faster frame rate (FPS) of 16.1 compared to Mask R-CNN, which scored 29.3 and 6.3 FPS, respectively.

## 5. Conclusions

This paper introduces an innovative deep learning method for Human Action Recognition (HAR) and musical note recognition in music performances, employing a Spatial Temporal Attention Graph Convolutional Network (STA-GCN). To facilitate this research, we carefully constructed a comprehensive dataset using advanced sensor technology, capturing precise hand keypoint data and instrument segmentation details. These sensor-derived datasets were essential for capturing the spatial and temporal dynamics crucial for our STA-GCN model. Our two-stage approach demonstrates the value of detailed, sensor-based data collection in enhancing the model’s ability to discern intricate patterns in HAR and music performance, contributing to advancements in the field of machine learning.

However, the STA-GCN model might be computationally expensive, potentially limiting its real-time applicability in some scenarios. Future work could explore lighter-weight model architectures or optimize the existing model for efficiency to address this limitation. Additionally, the current work focuses on the specific instrument, Morin khuur. Adapting the method to handle other instruments with different playing styles and techniques would necessitate further research and adjustments to the model.

## Figures and Tables

**Table 1:** The comparison with MediaPipe, OpenPose and MMPose.

Method	FPS	Incorrect	Missing
MediaPipe (a)	12	16	43
OpenPose (b)	1	24	9
MMPose (c)	4	0	0

**Table 2:** The standard scale length values of the instrument.

Size (cm)/Index of Fret	1	2	3	4	5	6	7	8	9	10	11	12	13	14
Large (56.6)	6	10.8	13.7	18.2	22.5	26.3	28.5	30.9	33.4	36.3	37.8	39.4	41.3	42.9
Medium (54)	5.7	10.3	13.2	17.6	21.5	25	27.2	29.7	32	34.4	35.8	37.6	39.2	40.6
Small (<54)	5.4	9.6	11.8	16.3	19.9	23.4	25.1	27.9	30.3	32.5	33.9	35.2	36.8	37.9

**Table 3:** The comparison of our proposed method performance with different data types.

Method	Accuracy	Loss
ST-GCN motion data	0.73	1.14
ST-GCN w/RGB	0.70	1.33
STA-GCN w/two camera data	0.43	2.76
STA-GCN wo/side camera data	0.67	2.55
STA-GCN wo/front camera data	0.61	2.13
STA-GCN motion data	0.81	0.93
STA-GCN w/RGB	0.72	0.96
STA-GCN motion data w/RGB	0.63	1.76

**Table 4:** A comparison of Mask R-CNN and YOLACT segmentation performance.

Method	AP_mask_	FPS
MASK-RCNN	29.3	6.3
YOLACT	32.6	16.1

## Data Availability

The data supporting the findings of this study are provided upon request by contacting the corresponding author due to author privacy concerns.
